# Perioperative cardiovascular monitoring of high-risk patients: a consensus of 12

**DOI:** 10.1186/s13054-015-0932-7

**Published:** 2015-05-08

**Authors:** Jean-Louis Vincent, Paolo Pelosi, Rupert Pearse, Didier Payen, Azriel Perel, Andreas Hoeft, Stefano Romagnoli, V Marco Ranieri, Carole Ichai, Patrice Forget, Giorgio Della Rocca, Andrew Rhodes

**Affiliations:** Department of Intensive Care, Erasme Hospital, Université Libre de Bruxelles, 808 route de Lennik, 1070 Brussels, Belgium; AOU IRCCS San Martino-IST, Department of Surgical Sciences and Integrated Diagnostics, University of Genoa, Largo Rosanna Benzi 8, 16132 Genoa, Italy; Adult Critical Care Unit, Royal London Hospital, Whitechapel Road, London, E1 1BB UK; Department of Anesthesiology and Critical Care, Lariboisière Hospital, Assistance Publique-Hôpitaux de Paris, University of Paris 7 Denis Diderot, 75475 Paris, Cedex 10 France; Department of Anesthesiology and Intensive Care, Sheba Medical Center, Tel Aviv University, Tel Aviv, 52621 Israel; Department of Anesthesiology and Intensive Care Medicine, University of Bonn, Sigmund-Freud-Str. 25, 53105 Bonn, Germany; Department of Human Health Sciences, Section of Anesthesiology and Intensive Care, University of Florence, Azienda Ospedaliero-Universitaria Careggi, Largo Giovanni Alessandro Brambilla 3, 50139 Florence, Italy; Department of Anesthesia and Intensive Care Medicine, University of Turin, S.Giovanni Battista Molinette Hospital, 10126 Turin, Italy; Medico-Surgical Intensive Care Unit, Saint-Roch University Hospital, University of Nice, 5 Rue Pierre Dévoluy, 06006 Nice, France; Service d’Anesthésiologie, Cliniques Universitaires Saint-Luc, Institute of Neuroscience (IoNS), Université catholique de Louvain, Avenue Hippocrate 10, 1200 Brussels, Belgium; Department of Anesthesia and Intensive Care Medicine, University Hospital, Medical School, University of Udine, P. le S. Maria della Misericordia 15, 33100 Udine, Italy; Department of Intensive Care Medicine, St George’s Healthcare NHS Trust, Blackshaw Road, London, SW17 0QT UK

## Abstract

A significant number of surgical patients are at risk of intra- or post-operative complications or both, which are associated with increased lengths of stay, costs, and mortality. Reducing these risks is important for the individual patient but also for health-care planners and managers. Insufficient tissue perfusion and cellular oxygenation due to hypovolemia, heart dysfunction or both is one of the leading causes of perioperative complications. Adequate perioperative management guided by effective and timely hemodynamic monitoring can help reduce the risk of complications and thus potentially improve outcomes. In this review, we describe the various available hemodynamic monitoring systems and how they can best be used to guide cardiovascular and fluid management in the perioperative period in high-risk surgical patients.

## Introduction

An estimated 230 million surgical procedures are performed each year around the world [1], and a significant number are in patients at risk of intra- or post-operative complications or both. Although less than 15% of in-patient procedures are performed in high-risk patients, such patients account for 80% of deaths [2-4]. Even for those patients who survive to leave hospital, post-operative complications remain an important determinant of functional recovery, long-term survival [5], and health-care costs. Thus, mitigation of these risks is important not only for the individual patient but also for health-care managers.

The risk of perioperative complications is related to various factors, including patient status and comorbidities, the type of surgery performed and its duration, the degree of urgency, the skills and experience of the operating and anesthetic teams, and the post-operative management. Insufficient tissue perfusion and cellular oxygenation due to hypovolemia, heart dysfunction or both is one of the leading causes of perioperative complications and poor outcomes [6-9]. Thus, effective fluid management to prevent and treat hypo/hypervolemia and titration of vasoactive drugs for heart dysfunction are crucial to maintain adequate oxygen delivery (DO_2_) and prevent fluid overload and its consequences [10-12]. Therefore, selecting the most appropriate hemodynamic monitoring device (for diagnosis and to guide therapies) may be an important first step in reducing the risk of complications. The aims of this review are to describe the available hemodynamic monitoring systems and to evaluate the most appropriate clinical setting for each.

## Basic hemodynamic monitoring

Clinical examination remains an important initial step in the hemodynamic assessment of high-risk surgical patients. However, individual vital signs often lack the specificity and sensitivity that are needed to guide hemodynamic management. For example, blood pressure is a variable influenced by both cardiac output (CO) and vascular tone; hence, blood pressure can remain within the normal range in the presence of low-flow states, including hypovolemia, as a result of increased peripheral vascular resistance. Similarly, heart rate may fail to reflect the development of hypovolemia under anesthesia [13].

Combining and integrating parameters from various hemodynamic monitoring systems may help improve our understanding of hemodynamic status [14]. For example, the combination of arterial pressure and the partial pressure of end-tidal carbon dioxide (PetCO_2_) can help differentiate between vasodilation and low CO as a cause of hypotension (PetCO_2_ transiently decreases when CO decreases) and may prevent ‘reflex’ fluid administration whenever blood pressure decreases. Similarly, a reduction in the PetCO_2_ value for the same minute ventilation (in the absence of hypothermia) suggests decreased pulmonary blood flow (and thus CO) and may serve as a trigger for more advanced hemodynamic monitoring.

### Arterial pressure

Continuous invasive measurement of arterial pressure helps identify the rapid fluctuations in arterial pressure that may occur in high-risk patients. Artifacts (over- or under-damping) should be carefully identified and eliminated, especially when systolic-diastolic components and waveform have to be analyzed. Non-invasive techniques for continuous measurement of blood pressure are usually performed in peripheral arteries and may become unreliable in case of vasoconstriction or low peripheral flow. Non-invasive assessment of pressure waveforms from more central measurement sites, such as the brachial artery, may be a valuable option in the future.

### Central venous pressure

A central venous catheter (CVC) is often used for administration of fluids, vasopressors, and inotropes and for measurement of central venous pressure (CVP). Since transmural CVP is the only value related to right ventricular (RV) preload but is not commonly monitored, interpretation of CVP values must take into account intrathoracic pressure changes, which are largely influenced by mechanical ventilation. Thus, changes in CVP with concomitant CO variations give an indication of RV function and potential peripheral venous congestion, the latter of which is an important factor for organ perfusion [15]. In addition, careful checking of the CVP wave may help to diagnose tricuspid regurgitation with a ‘v’ wave during systole. When the CVP is low (<6 mm Hg) with a concomitant low CO, there is almost certainly some degree of hypovolemia. Although changes in CVP correlate poorly with changes in CO [16] (as for pulmonary artery occlusion pressure), they can be used to assess the dynamic response to a fluid challenge [17] and to diagnose severe hypovolemia or cardiac dysfunction or both, especially where other monitoring systems are not available.

## Cardiac output monitoring

The perioperative period is characterized by large variations in whole body oxygen consumption (VO_2_). The main goal in this period is to maintain an adequate DO_2_ to meet the fluctuating tissue oxygen requirements. Global DO_2_ is determined by CO and the oxygen content of arterial blood, and so after correction of hypoxemia and anemia (topics that will not be dealt with here), maintenance of an adequate CO is the next logical step to improve DO_2_. There are various methods available for monitoring CO [18], although a survey indicated that CO is routinely monitored in high-risk surgical patients by only about 35% of practitioners in Europe and North America [19] (Table 1).

**Table 1:** **What hemodynamic monitoring do you routinely use for the management of high-risk surgery patients? (Please mark all that apply)**

	**ASA respondents**	**ESA respondents**
	**(n = 237)**	**(n = 195)**
**Answer options**	**Response percentage**	**Response percentage**
Invasive arterial pressure	95.4%	89.7%
Central venous pressure	72.6%	83.6%
Non-invasive arterial pressure	51.9%	53.8%
Cardiac output	35.4%	34.9%
Pulmonary capillary wedge pressure	30.8%	14.4%
Transesophageal echocardiography	28.3%	19.0%
Systolic pressure variation	20.3%	23.6%
Plethysmographic waveform variation	17.3%	17.9%
Pulse pressure variation	15.2%	25.6%
Mixed venous saturation (ScvO_2_)	14.3%	15.9%
Central venous saturation (SvO_2_)	12.7%	33.3%
Oxygen delivery (DO_2_)	6.3%	14.4%
Stroke volume variation	6.3%	21.5%
Near-infrared spectroscopy	4.6%	5.1%
Global end-diastolic volume	2.1%	8.2%

From [19]. ASA, American Society of Anesthesiology; ESA, European Society of Anaesthesiology.

### Doppler echocardiography

Though difficult to use as a continuous monitor of CO with conventional probes, transthoracic (TTE) or transesophageal (TEE) echocardiography can provide immediate point-of-care assessment of acute hemodynamic changes in selected patients. Echo techniques can also help to visualize the lungs, but this is beyond the scope of this review. Obviously, it is not possible to use TEE in all types of surgery. In addition to the estimation of CO (usually easier with TEE than with TTE), Doppler echocardiographic examination can provide an indication of cardiac function because it allows visualization of the cardiac chambers, valves, and pericardium [20]. It also allows measurement of the ejected stroke volume (SV) and derived left ventricular (LV) function parameters.

TEE provides several views, including the following:

- The LV short-axis view, which can be used to evaluate LV function. Calculation of the LV fractional area contraction, or the simpler ‘eyeballing method’, informs about the kinetic (contractile) state and the shape (volume) of the heart. Poor contractility may indicate that inotropic support could help, and ‘kissing’ of the papillary muscle may indicate the need for fluids if the right heart is functioning normally. The short-axis view may also be used to identify septal dyskinesia. The finding of an RV D-shape may suggest the presence of RV dysfunction/failure, indicating a non-adaptation to an acute increase in RV afterload (pulmonary embolism) or RV myocardial ischemia.
- The four-chamber view, which can help in assessing LV and RV function by evaluation of the right-to-left size ratio (normal <0.6).

In more advanced echocardiographic evaluation, fluid status and fluid responsiveness can also be assessed in mechanically ventilated patients by means of the superior vena cava collapsibility index (TEE bicaval view) or inferior vena cava distensibility index (TTE subcostal view). In addition, echocardiography allows the rapid and reliable estimation of SV. Finally, there are particular and specific conditions in which diagnosis and treatment are strictly related to the echocardiographic examination (for example, pericardial effusion, valve disruptions, aortic dissection, and systolic anterior motion of the mitral valve).

A miniaturized, disposable monoplane TEE probe that can be left in place for up to 72 hours (ClariTEE™, ImaCor Inc., Garden City, NY, USA) was recently introduced and has the potential to provide ongoing qualitative cardiac assessment [21]. We believe that, where expert echocardiography skills are not available, training programs should be developed to ensure that clinicians taking care of the high-risk patient are familiar with at least the basic applications of TTE and TEE.

### Pulmonary artery catheter

Though criticized in recent years for its intrinsic invasiveness and no clear evidence of improved outcomes [22-25], the pulmonary artery catheter (PAC) is the only tool that provides continuous monitoring of pulmonary artery pressure, right-sided and left-sided filling pressures, CO, and mixed venous oxygen saturation (SvO_2_). Although the PAC can now be largely replaced by less invasive hemodynamic monitoring techniques in many cases, in some complex clinical situations (for example, cardiac surgery, organ transplant surgery, and surgery associated with major fluid shifts or high risk of respiratory failure or in patients with compromised RV function), the PAC still represents a valuable tool when used by physicians adequately trained to correctly interpret and apply the data provided [26,27]. In such patients, the PAC can be inserted for limited periods of time and removed when no longer necessary.

### Other cardiac output monitoring devices

#### Pulse contour analysis

SV can be estimated continuously by analysis of the arterial pressure waveform, usually derived from an indwelling arterial catheter or by a non-invasive finger pressure cuff. To calculate SV from a pressure trace, the algorithms used by these devices have to compensate for the overall impedance of the system on the basis of the estimation of compliance and resistance of the cardiovascular tree. In this regard, optimization of the input signal is imperative, and severe distortions of the arterial waveform (for example, severe arrhythmias and multiple ectopic beats) and inadequate response of fluid-filled transducer systems (that is, over- and under-damping) [28] can result in unreliable CO measurement.

##### Calibrated devices

- The PiCCOplus™/PiCCO_2_™ system (Pulsion Medical Systems, Munich, Germany) consists of a thermistor-tipped catheter which is usually placed in the femoral artery, although catheters for radial, axillary, or brachial applications are also available. The PiCCO™ device measures CO by transpulmonary thermodilution, which additionally provides the computation of volumetric preload parameters—global end-diastolic volume (GEDV) and intrathoracic blood volume—and extravascular lung water (EVLW). The CO measured by the Stewart-Hamilton principle from the thermodilution curve is used to calibrate a pulse contour algorithm, which measures the area under the systolic pulse pressure curve and calculates the SV in order to provide beat-by-beat CO measurement. The system has to be frequently recalibrated, at least every 8 hours in hemodynamically stable patients and more often if changes in vasoactive support are provided [29]. The system has been validated in a variety of clinical settings [30].
- The EV1000™/VolumeView™ system (Edwards Lifesciences, Irvine, CA, USA) has been more recently introduced and is analogous to the PiCCO™ monitor, using pulse wave analysis to calculate CO. A proprietary thermistor-tipped femoral artery catheter and a separate sensor are the main components of the system. This system requires calibration by transpulmonary thermodilution. It has been validated against the PiCCO™ and transpulmonary thermodilution in critically ill patients [31].
- The LiDCO™*plus* system (LiDCO Ltd, Cambridge, UK) uses pulse power analysis to calculate SV and therefore is not technically a pulse contour device. The algorithm is based on the principle of conservation of mass (power), assuming a linear relationship between the net power change and the net flow in the vascular system. This system requires correction for vascular compliance, with calibration using a transpulmonary lithium indicator dilution technique performed via an indwelling arterial catheter. It has been validated in critically ill patients [32,33].

##### Uncalibrated devices (without external calibration)

With preloaded data

- The PulsioFlex™ system (Pulsion Medical Systems) displays trends of estimated CO by using the patient’s anthropometric and demographic characteristics (necessary for internal calibration), analysis of the arterial pressure tracing, and a proprietary algorithm for data analysis. The system requires a dedicated additional sensor, which can be connected to a regular arterial pressure catheter. Based on the same pulse contour algorithm used by the PiCCO™, the device can be calibrated by entering a CO obtained from an external source (for example, Doppler echocardiography) or by the system’s own internal algorithm.
- The LiDCO™*rapid* (LiDCO Ltd) device uses the same algorithm as the LiDCO™*plus* system, but instead of lithium dilution, nomograms based on the patient’s age, weight, and height are used to estimate SV and CO (so-called ‘nominal’ SV and CO). An externally estimated CO can be used to calibrate the device.
- The FloTrac™/Vigileo™ system (Edwards Lifesciences) consists of a proprietary transducer (FloTrac™) connected to a standard (radial or femoral) arterial catheter. Individual demographic variables (age, sex, height, and weight) and a database containing CO variables derived by using the PAC are used to calculate impedance and a ‘normal’ SV against which the standard deviation of the pulse pressure sampled during a 20-second interval is correlated to estimate CO. Arterial waveform analysis is used to calculate vascular resistance and compliance. The algorithm used by the Vigileo™ device has been modified over time, and recent studies evaluating the device in the perioperative setting have shown an improved performance and a significant reduction in the time needed to adapt to vascular dynamics. In the intensive care unit (ICU) setting, concerns remain regarding the accuracy in situations of acute hemodynamic instability as well as hyperdynamic conditions, although recent software modifications seem to improve the reliability of CO measurements. The FloTrac™/Vigileo™ system has been shown to be suitable for integration into perioperative optimization protocols, resulting in improved clinical outcomes [34,35].

Without preloaded data

- The MostCare system (Vytech, Padua, Italy), powered by the pressure recording analytical method, performs a beat-to-beat estimation of SV and CO by analyzing the pressure waveform, sampled at high resolution (1,000 points per second = 1 kHz). The area under the pressure wave is determined during the whole cardiac cycle. In each phase, the method identifies specific points (‘points of instability’) characterized by modifications in velocity and acceleration in relationship to the previous and the subsequent point. All of these ‘points of instability’, mainly caused by reflected waves from the periphery (backward travelling waves), give the arterial pulse its specific profile, which is analyzed by MostCare for estimation of the vascular impedance (Zt). The contribution of the reflected waves to the forward travelling wave can be accurately identified only with a very high sampling rate. The ability to update the Zt during each heart beat makes the system extremely reactive when abrupt changes in impedance occur (for example, changes in vascular tone) [36,37]. Although some promising clinical data are available [38], larger validation studies are needed to confirm these observations. A multicenter study comparing MostCare with echo-Doppler for CO measurement was recently completed (ClinicalTrials.gov identifier: NCT01678950).

##### Non-invasive pulse contour analysis

- Recently, several newly marketed monitors have purported to track changes in arterial pressure non-invasively from finger probes. These include the continuous non-invasive arterial pressure probe (used with the LiDCO™ system) and the ClearSight device (Edwards Lifesciences). Based on the volume-clamp technique of Penaz [39] incorporated into a finger cuff, a brachial artery pressure waveform is reconstructed. The ClearSight monitor estimates CO on a beat-to-beat basis by dividing the integrated pulsatile systolic area by the aortic input impedance, which in turn is derived from a three-element Windkessel model incorporating patient-specific aortic mechanical characteristics [40]. Preliminary clinical validation studies, currently limited to a critical care setting and cardiac surgery, have shown acceptable agreements in CO when compared with thermodilution measurements performed with a PAC [41-44]. These monitors have the potential to track SV and CO in situations requiring early hemodynamic intervention when more invasive monitoring modalities are not readily available.

#### Doppler monitoring devices

- Esophageal Doppler offers a minimally invasive determination of CO. The CardioQ™/CardioQ-ODM™ (Deltex Medical Ltd, Chichester, UK) is the most commonly used device. Esophageal probes measure blood flow in the descending part of the aorta. SV is calculated by multiplying the cross-sectional area of the aorta (from nomograms based on height, weight, and age) by the blood flow velocity. Technical and methodological concerns regarding probe positioning and the use of nomograms have been raised.
- A transthoracic continuous Doppler CO monitor, the USCOM (Uscom, Sydney, Australia), is also available for intermittent SV and CO assessment. Using supra- or parasternal windows, flow velocity at the level of the aortic or pulmonary valves can be assessed non-invasively. Aortic diameter can be loaded from another measurement (two-dimensional echocardiography) or from a nomogram as mentioned above. The technique is rapid and can be used in adult and pediatric patients. Clinical validation studies have shown conflicting results, partially due to variations in signal acquisition inherent in the technique [45].

#### Applied Fick principle and dye dilution

- The NICO™ System (Novametrix Medical Systems, Wallingford, CT, USA), using the partial CO_2_ rebreathing method, applies the Fick principle to CO_2_ in patients who are intubated and mechanically ventilated via a disposable rebreathing circuit that is added to the ventilator tubing. CO_2_ production is calculated as the product of CO_2_ concentration and airflow during a breathing cycle, and arterial CO_2_ content is derived from end-tidal CO_2_ and its corresponding dissociation curve. The rebreathing loop can induce an intermittent partial rebreathing state in 3-minute intervals. This rebreathing cycle results in an increased end-tidal CO_2_, mimicking a decrease in CO_2_ production. The differences in these values can be used to calculate CO. The system is not really clinically acceptable, because the assessment of CO is possible only in patients with fixed ventilator settings and good respiratory function with no relevant pulmonary shunt [46,47].
- DDG-30® pulsed dye densitometry (Nihon Kohden, Tokyo, Japan) is based on the transpulmonary dye dilution technique using indocyanine green (ICG). Signal detection in the arterial blood is performed by transcutaneous optical absorbance measurements similar to pulse oximetry. CO is calculated from the ICG-dye dilution curve according to the Stewart-Hamilton principle. Factors that compromise signal detection, such as vasoconstriction, interstitial edema, movement, or ambient light artifacts, all limit the reliability of CO assessment using this method [48].

#### Bioimpedance and bioreactance

- Electrical bioimpedance estimates CO continuously by detecting variations in thoracic or whole body impedance induced by cyclic changes in blood flow. Electrodes attached to the skin (BioZ, CardioDynamics, San Diego, CA, USA) or an endotracheal tube (ECOM™, Conmed Corporation, Utica, NY, USA) provide electric current stimulation, and signal variations are analyzed by using mathematical algorithms. The reliability of these systems is poor [49,50].
- The Bioreactance® technique (NICOM®, Cheetah Medical Ltd, Maidenhead, Berkshire, UK) analyzes the variations in the frequency spectra associated with delivery of an oscillating electrical current. This technique may be somewhat superior to the bioimpedance technique [51,52] but is dependent on body size [53] and the aeration of the lung. It is less accurate in diseased lungs in which reactance may be affected by the amount of EVLW and alveolar collapse or consolidation or both.

### Pitfalls in the interpretation of cardiac output

Although CO can be measured with reasonable accuracy and precision with some of these systems, it is difficult to assess the optimal CO for an individual patient. A ‘normal’ or even high CO does not preclude the presence of inadequate regional and microcirculatory flow, and a low CO may be adequate in a context of low metabolic demand, especially during surgery under general anesthesia. Moreover, simple identification of a low CO does not tell us what to do about it. To correctly interpret the data acquired by any of the described devices, we need to combine/integrate several variables to help decide whether the CO/SV is adequate and how it can be optimized in the most effective manner.

### How to select the best system

All monitoring systems have unique characteristics in terms of accuracy, precision, validity, stability, and reliability [18]. Not all monitoring devices have been evaluated against the same set of criteria, and uncertainty remains regarding acceptance thresholds for the performance of CO monitors and the used reference techniques [54-57]. Clinicians must consider the technical limitations of each monitoring system and the potential trade-off between more invasive but highly accurate measurements of CO and less invasive but also less accurate modalities.

**Figure 1:**
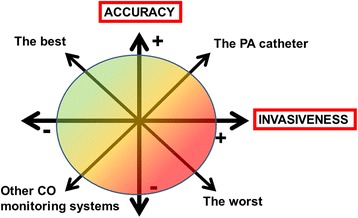
The compromise between accuracy and invasiveness of monitoring systems. CO, cardiac output; PA, pulmonary artery.

**Figure 2:**
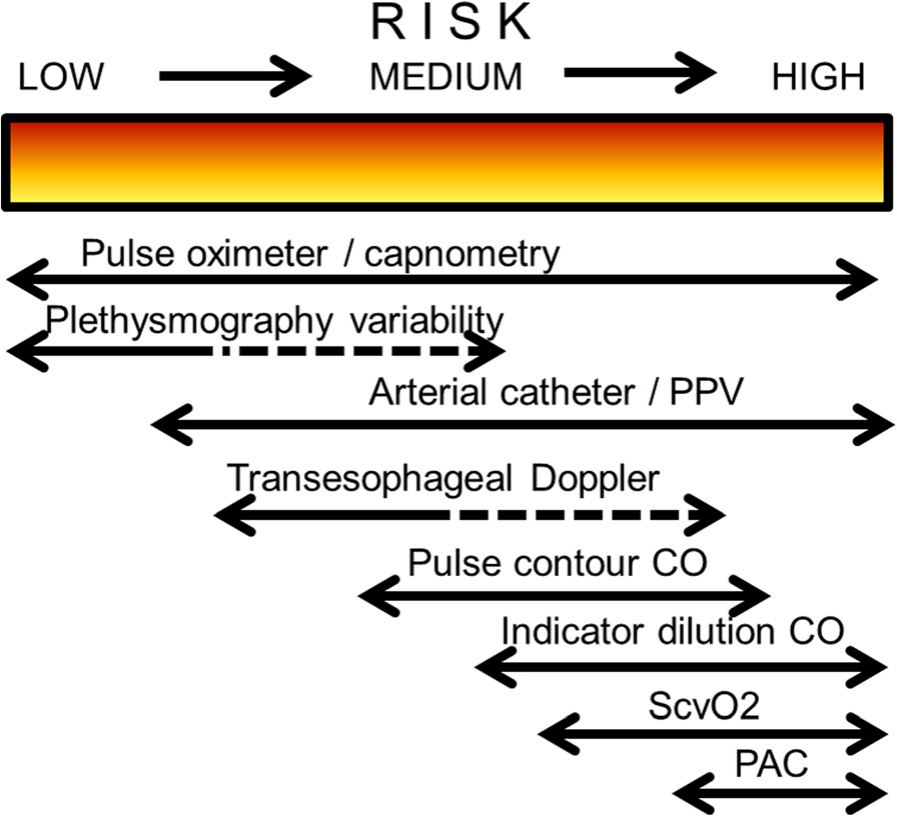
Possible choice of monitoring system in relation to a patient’s degree of perioperative risk. CO, cardiac output; PAC, pulmonary artery catheter; PPV, pulse pressure variation; ScvO_2_, central venous oxygen saturation.

Several questions can be raised when considering choice of CO monitoring in the perioperative period:

1. Are we ready to accept a less accurate measurement in order to limit invasiveness? (Figure 1). A less accurate measurement may be acceptable if the trend analysis is reliable. Cost may also be an important issue.
2. Do we need continuous, semi-continuous, or intermittent measurements? Most complications after surgery do not have a sudden onset (except sudden cardiac failure due, for example, to myocardial infarction or pulmonary embolism) or an obvious cause (for example, massive bleeding during surgery) but develop slowly; therefore, semi-continuous or intermittent measurements may be acceptable. However, it should be noted that only beat-by-beat measurement of SV allows assessment of the response to preload-modifying maneuvers, such as a fluid challenge or passive leg raising (PLR) test.
3. Are calibrated or uncalibrated systems preferable? Non-calibrated systems are acceptable for the operating room (OR) or the post-anesthesia care unit (PACU) but may not be suitable for more complex cases, especially in the ICU. In unstable patients, there is a necessity to ‘re-calibrate’ more often because of frequent changes in vascular tone and also because derived variables (for example, EVLW and GEDV) need to be re-calculated. A practical option may be to use an uncalibrated system in the OR/PACU and replace it with a calibrated system in the ICU.
4. What alarms do we need? A major problem for patient surveillance by telemetric monitoring is artifact robustness. Any system with too many false alarms is prone to failure as personnel become desensitized.
5. What kind of monitoring for what kind of patient? This is not a ‘one size fits all’ decision; rather, the optimal monitoring technique for each patient will vary depending on the degree of risk and the extent of the surgical procedure (Figure 2).

## Fluid management

**Figure 3:**
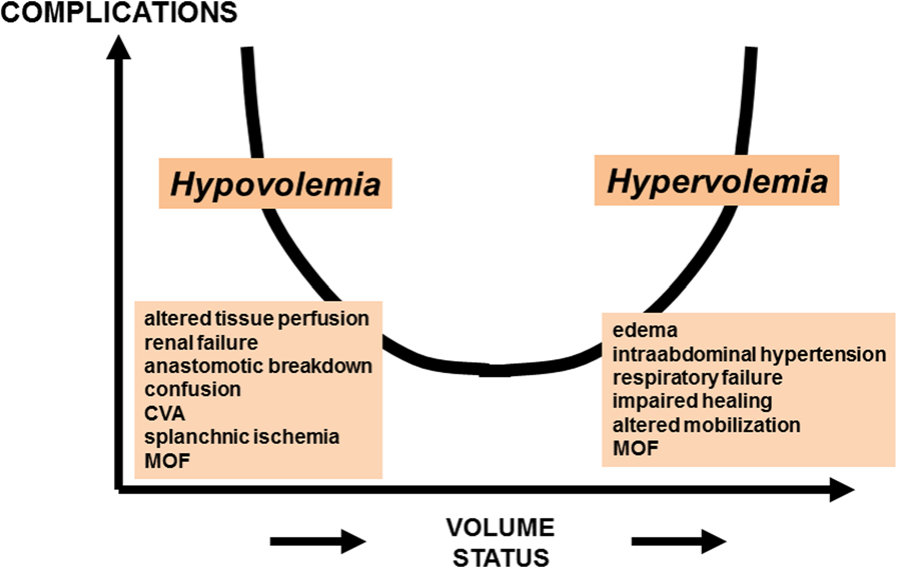
Both hypo- and hypervolemia are associated with more complications. CVA, cerebrovascular accident; MOF, multiple organ failure.

Inadequate fluid management may lead to reduced CO and DO_2_ to injured tissues, which is associated with an increased incidence of post-operative complications. Moreover, the systemic inflammatory response associated with tissue injury results in capillary leak and tissue edema. Fluid restriction and diuresis may decrease edema in patients with poor ventricular function but may also increase the incidence of acute kidney injury. Meanwhile, excessive fluid administration may lead to a range of adverse effects, including coagulopathy and edema of lungs, gut, and peripheral tissues (Figure 3). Retention of sodium and water following surgery may reduce fluid requirements. Once the patient is stabilized, fluids should be given only to correct deficit or continuing losses. Unfortunately, estimates of fluid deficit that are based on traditional physiological parameters, such as heart rate, blood pressure, and cardiac filling pressures, are not sufficient.

### Static indicators of preload

- CVP: Many high-risk surgical patients have a CVC in place, and a CVC is a requirement for some devices needing calibration by thermodilution. Despite its limitations (vide supra), changes in CVP over time may be helpful to guide fluid therapy, especially when the CVP is low and associated with low flow. A CVP of more than 8 mm Hg might also be considered an ‘alarm’ for potential venous congestion associated (or not) with fluid overload [15].
- GEDV and EVLW: These are volumetric parameters derived from transpulmonary thermodilution and are integrated into the PiCCO™*plus*, PiCCO_2_™, and EV1000™ monitors. EVLW can help in the identification of (cardiogenic or non-cardiogenic) pulmonary edema and has the potential to increase the safety of fluid therapy in patients with structural lung disease, acute respiratory distress syndrome, or congestive heart failure.
- The end-diastolic area of the left ventricle may be the most reliable static parameter of preload but is largely dependent on LV diastolic compliance. Its ability to accurately predict fluid responsiveness is limited.

### Functional hemodynamic parameters

Positive pressure ventilation induces cyclical changes in intrathoracic pressure, which affect preload by decreasing venous return to the right heart and increasing venous return to the left ventricle. The degree of the resulting changes in LV SV (stroke volume variation; SVV) and pulse pressure (pulse pressure variation; PPV) better predict fluid responsiveness than do static parameters, when RV function is not a limitation and for a fixed tidal volume. Most devices using pulse contour analysis, including the current version of the non-invasive ClearSight monitor, display SVV and PPV. Despite the numerous validity criteria required to interpret such variations, these variables may help predict fluid responsiveness at different thresholds and have been integrated into hemodynamic optimization protocols [58].

Respiratory variations in the pulse oximeter plethysmographic waveform have been shown to predict fluid responsiveness in mechanically ventilated patients, similar to changes in the arterial pressure waveform [59]. The Masimo™ device (Masimo Corporation, Irvine, CA, USA) provides automated calculation of the pleth variability index (PVI) by measuring changes in perfusion index over a time interval including at least one complete respiratory cycle. The PVI has been shown to predict fluid responsiveness in various perioperative settings and has been integrated into fluid optimization algorithms. However, the PVI has the same limitations as the other dynamic parameters and has limited accuracy in the presence of vasoconstriction with or without the use of vasopressors [60-62].

#### Limitations

It is important to note that all of the dynamic variables have significant confounding factors [58]. The reliability of these indices is affected by spontaneous breathing activity, arrhythmias, right heart failure, decreased chest wall compliance, and increased intra-abdominal pressure, although most of these limitations are uncommon in the OR. Nevertheless, in the ICU, a relatively small proportion of patients present suitable criteria for these indices [63]. Another major limitation of dynamic parameters is that they are dependent on the size of the tidal volume. Some authors have suggested that they require a tidal volume of at least 8 mL/kg body weight [64], although they have been successfully used with tidal volumes of 6 to 8 mL/kg body weight [61,62]. A recent study and meta-analysis have indicated a decreased rate of post-operative complications when low tidal volumes are applied during anesthesia [65,66], and increased use of protective ventilation (lower tidal volumes) in the OR may reduce the usefulness of dynamic parameters or at least require new interpretation rules. Finally, within a range of PPV values of 9% to 13%, fluid responsiveness cannot always be reliably predicted; there is a ‘gray zone’ in which prediction of fluid responsiveness is difficult. One study [67] indicated that fluid responsiveness could not be reliably predicted by using dynamic measures in as many as 25% of anesthetized patients.

A PLR test has been suggested to overcome some of these limitations in dynamic evaluation but should be performed rigorously with simultaneous analysis of continuous CO monitoring. It is obviously impractical during most operative conditions [68]. In addition, the blood volume shift from the leg to the central compartment is non-predictable. In a hypovolemic state, it is reasonable to consider this volume shift less than that generated in ‘normal’ volemic conditions.

Despite these limitations and confounding factors, whenever possible, one is advised to assess fluid responsiveness by using the available functional hemodynamic parameters before attempting to increase CO with fluid administration. This approach can indicate if and when CO can be further increased by fluids and can identify when the flat portion of the cardiac function curve has been reached, thus preventing unnecessary fluid loading [58]. It is important to remember that, generally speaking, fluid responsiveness is not an (absolute) indication to give fluids. Decisions about fluid administration should be based not only on dynamic parameters but also on the likely risk associated with fluid administration. During surgery, systematic fluid administration in the presence of fluid responsiveness may improve post-operative outcomes [69].

## Mixed venous oxygen saturation

Changes in SvO_2_ may reflect important pathophysiological changes in the relationship between DO_2_ and VO_2_, both of which may fluctuate significantly during the perioperative period.

Reorganization of the Fick equation shows that:

$$ \mathrm{S}\mathrm{v}{\mathrm{O}}_2 = \mathrm{S}\mathrm{a}{\mathrm{O}}_2\hbox{--}\ \left(\mathrm{V}{\mathrm{O}}_2/\left[\mathrm{C}\mathrm{O}\times \mathrm{H}\mathrm{b}\times \mathrm{C}\right]\right), $$

where C is the amount of oxygen bound to 1 g of hemoglobin (Hb). From this equation, it is clear that SvO_2_ will decrease in the presence of hypoxemia, hypermetabolic states (increased VO_2_), a decrease in CO, or anemia. Therefore, changes in SvO_2_ are directly proportional to those in CO but only when arterial oxygen saturation (SaO_2_), VO_2_, and Hb concentration remain constant. The SvO_2_ is around 75% in healthy patients but is closer to 70% in acutely ill patients who have a somewhat lower Hb concentration.

Central venous oxygen saturation (ScvO_2_) is used as a surrogate for SvO_2_ when a PAC is not *in situ*, but has some limitations. Although the determinants of ScvO_2_ and SvO_2_ are similar, they cannot be used interchangeably [70-73]. Regional variations in the balance between DO_2_ and VO_2_ result in differences in the Hb saturation of blood in the superior and inferior vena cavae [74]. ScvO_2_ is affected disproportionately by changes in the upper body and does not reflect the SvO_2_ of coronary sinus blood [74]. In healthy individuals, ScvO_2_ may be slightly less than SvO_2_ [75] because of the high oxygen content of effluent venous blood from the kidneys [76], but this relationship is reversed during periods of hemodynamic instability as blood is redistributed to the upper body at the expense of the splanchnic and renal circulations [77]. In shock states, therefore, ScvO_2_ may exceed SvO_2_ by up to 20% [72]. This lack of equivalence has been demonstrated in various groups of acutely ill patients, including not only those with shock [70,71,78] but also patients undergoing general anesthesia for cardiac [73,79] and non-cardiac [71,80] surgery. Even trends in ScvO_2_ do not closely reflect those of SvO_2_ [70,73,78].

Lower values of ScvO_2_ have been associated with more complications in patients undergoing cardiothoracic surgery [81]. Therefore, some authors have proposed to maintain SvO_2_ or ScvO_2_ above a cutoff value. In patients undergoing elective cardiac surgery, administration of intravenous fluid and inotropic therapy to attain a target SvO_2_ of at least 70% in the first 8 hours after surgery was associated with fewer complications and a shorter hospital stay [82]. In patients undergoing major abdominal (including aortic) surgery, achieving an oxygen extraction ratio of less than 27% (from intermittent measurements of ScvO_2_) was associated with a shorter hospital stay [83].

During surgery, this measurement is less informative: firstly, hypoxemia is generally corrected; secondly, under anesthesia, especially with neuromuscular paralysis, oxygen use decreases in all tissues, so that reductions in ScvO_2_ are uncommon [84]. Nevertheless, low ScvO_2_ values imply first and foremost that CO may be inadequate. At the same time, very high ScvO_2_ values may imply that oxygen extraction is low, purporting a worse prognosis, at least during cardiac surgery [85].

## Blood lactate concentrations

Lactate is a physiological substrate (carbohydrate) produced from pyruvate reduction during cytosolic glycolysis. In stable conditions, lactate production and elimination are equivalent (that is, 1,200 to 1,500 mmol per day), leading to a stable blood lactate concentration of 0.8 to 1.2 mmol/L. The net flux of lactate depends on the difference between release and uptake and varies among organs and with their energetic conditions [86]. Hyperlactatemia is associated with increased morbidity and mortality in critically ill patients [87-90]. Persistent hyperlactatemia is a more relevant indicator of poor outcome than an isolated elevated lactate value is. Hyperlactatemia is not always a consequence of tissue hypoxia; sometimes, it stems from an accelerated ‘aerobic’ glycolysis resulting from cytokine influence and catecholamine stimulation, a situation termed ‘stress hyperlactatemia’. In practice, irrespective of the different metabolic modifications, an elevated lactate level indicates the presence of shock, and a decrease in lactate levels over time is a good indicator of effective treatment. Accordingly, repeated blood lactate measurements are recommended to monitor lactate production and clearance over time during surgery in high-risk patients.

## Management strategies based on perioperative monitoring

There is good evidence that use of flow-based hemodynamic monitoring combined with hemodynamic manipulation in the perioperative period can reduce morbidity and sometimes mortality [83,91-97]. For a variety of reasons, however, this approach has not been adopted everywhere and has even been challenged [98]. Indeed, there have been some important problems with many clinical trials in the field, such as lack of blinding and suboptimal management of the control group.

There are basically two options to optimize perioperative cardiovascular management (Table 2), both of which aim to increase SV/CO by means of fluid loading (increase in cardiac preload) or inotrope administration (increase in contractility) or both:

- One option is reactive, by applying a rapid intervention only when a hemodynamic change occurs. One should then individualize treatment with fluid challenge techniques. The response to the rapid administration of a fluid bolus (for example, 250 mL) can be evaluated during surgery (especially in the presence of signs of fluid responsiveness). The response can be monitored by evaluating the blood pressure or heart rate, but the CO/SV response is much more accurate. Inotropic agents are added in the absence of an adequate response.
- The other option is pro-active and is based on a strategy of hemodynamic manipulation targeting supranormal CO or DO_2_ values to minimize the risk of tissue hypoperfusion. Adequate fluid administration is the first element of this strategy. Several studies have indicated that fluid management based on PPV, SVV, and SV optimization may decrease post-operative wound infections and possibly post-operative organ dysfunction [99,100]. Inotropic agents may be added if fluids alone are not sufficient for this purpose. There is a risk of overtreatment as excessive use of dobutamine has been associated with increased rates of complications [101]. The use of dopexamine as an alternative has given controversial results [102,103].

**Table 2:** **Options to optimize perioperative hemodynamic management in high-risk patients**

**▪**	**Reactive**
	Correct hypotension, tachycardia.
	Give fluids in the presence of suspected hypovolemia with increased pulse pressure variation (PPV), systolic pressure variation, stroke volume variation (SVV), or pleth variability index (PVI).
	Identify a reduction in cardiac output and react promptly with fluid challenge.
	Identify a reduction in central venous oxygen saturation (ScvO_2_) and react promptly with fluid challenge.
**▪**	**Pro-active**
	Maintain arterial pressure and heart rate within acceptable ranges.
	Maximize stroke volume.
	Maintain PPV or SVV at less than 12% or PVI at less than 14%.
	Maintain cardiac index (CI) or oxygen delivery (DO_2_) in a desired range (for example, CI of more than 4.5 L/minute/m^2^ and DO_2_ of more than 600 mL/minute/m^2^).
	Maintain ScvO_2_ at more than 65%.

## Conclusions

Cardiovascular monitoring systems play an important role in optimizing perioperative hemodynamic management. Use of hemodynamic monitoring devices *per se* in the perioperative setting has not been linked to improved outcomes; however, appropriate measurement and interpretation of cardiovascular variables may help guide therapeutic interventions, which in turn can improve patient outcomes. The most appropriate system must be selected for the individual patient prior to surgery, taking into consideration the individual risks of the patient and the procedure. Appropriate interpretation of the information offered by hemodynamic monitoring requires the integration of several variables. Echocardiography is increasingly used as a first tool to identify a problem and help select initial treatment. To improve patient management and outcome, the clinician must understand the advantages and the limitations of the various tools and parameters used during perioperative care.

## Abbreviations

CO: Cardiac output
CVC: Central venous catheter
CVP: Central venous pressure
DO_2_: Oxygen delivery
EVLW: Extravascular lung water
GEDV: Global end-diastolic volume
Hb: Hemoglobin
ICG: Indocyanine green
ICU: Intensive care unit
LV: Left ventricular
OR: Operating room
PAC: Pulmonary artery catheter
PACU: Post-anesthesia care unit
PetCO_2_: Partial pressure of end-tidal carbon dioxide
PLR: Passive leg raising
PPV: Pulse pressure variation
PVI: Pleth variability index
RV: Right ventricular
ScvO_2_: Central venous oxygen saturation
SV: Stroke volume
SvO_2_: Mixed venous oxygen saturation
SVV: Stroke volume variation
TEE: Transesophagel echocardiography
TTE: Transthoracic echocardiography
VO_2_: Oxygen consumption
Zt: Vascular impedance
